# Supplementary material for: Research on the mechanism of multilayer spiral fog screen dust removal at the comprehensive excavation face
Source: PLoS One. 2022 Apr 14;17(4):e0266671. doi: 10.1371/journal.pone.0266671 (PMC9009714; doi:10.1371/journal.pone.0266671)
Supplement: Attachment — Submitted filename: Reviewers comments.pdf [file pone.0266671.s001.pdf]

This manuscript proposes a dust removal method using a multilayer spiral fog curtain with potential application in the dust-producing areas in mines. Considering the dangers of high-concentration dusts for miners, researches on this field are very essential. The manuscript includes experimental and theoretical sections. In the experimental section, an equipment has been made and dust removal rate and efficiency have been measured. In the theoretical section, numerical simulation of spray airflow and particle trajectory has been performed based on fluid mechanics relations. The manuscript is good in general. I recommend acceptance with minor comments as follows:

1. The manuscript file doesn't have page numbers and line numbers. It is hard to refer to a particular word or sentence when you cannot refer to line numbers.
2. Has the device been tested in a real mine environment?
3. In the present manuscript, numerical simulations investigate the fluid mechanics of the system and experimental section studies the efficiency of dust removal (mass transfer). For future studies, I recommend the authors to couple mass transfer relations with the fluid mechanics equations and predict the dust removal efficiency by numerical simulations.
4. In the introduction, authors should explain more about the innovation of their work.
5. It is recommended to add a Nomenclature table to the manuscript.
6. The quality and sharpness of some figures are not very good (for example: Figs. 10, 5 and 6).
7. In section 3.2, some equations don't have equation numbers.
8. In the text, reference number should be inserted after the "author's name et al.".
9. In the text, please use the same format to refer to the figures ("Figure" or "Fig. ").
10. Please use a space before the first bracket of all in-text citations.

11. The font of some parts of the manuscript is different from the rest of the text (such as references, some tables, the first paragraph of the conclusion, equation numbers and etc.). Please correct this problem in the manuscript.
12. Some parts of the text aren't justified (for example: references, the first paragraph of the section 3).
13. After all the headline numbers, the punctuation symbol is required. Some headlines also start with a lowercase letter that need to be corrected.
