# Supplementary material for: Research on the mechanism of multilayer spiral fog screen dust removal at the comprehensive excavation face
Source: PLoS One. 2022 Apr 14;17(4):e0266671. doi: 10.1371/journal.pone.0266671 (PMC9009714; doi:10.1371/journal.pone.0266671)
Supplement: Attachment — Submitted filename: Response to Reviewers.pdf [file pone.0266671.s002.pdf]

Dear Editors and Reviewers:

Thank you for your letter and for the reviewers' comments concerning our manuscript entitled "Research on the mechanism of multilayer spiral fog screen dust removal at the comprehensive excavation face". Those comments are all valuable and very helpful for revising and improving our pa per, as well as the important guiding significance to our researches. We have studied comments carefully and have made correction which we hope meet with approval. The paper is revised in word's revision mode, and the red area indicate the revised portion. The main corrections in the paper and the responds to the reviewer's comments are as flowing:

Response to Editor

1.The manuscript has complied with PLOS ONE style requirements, including file naming requirements.

2. Any text relating to funding has been removed from the manuscript.

3. Corresponding author has ORCID iD and validates it in Editorial Manager.

4. The reference list has been checked to ensure it is complete and correct.

Response to Reviewer#1

1. Fig 12 has been replaced with a clear picture in this manuscript

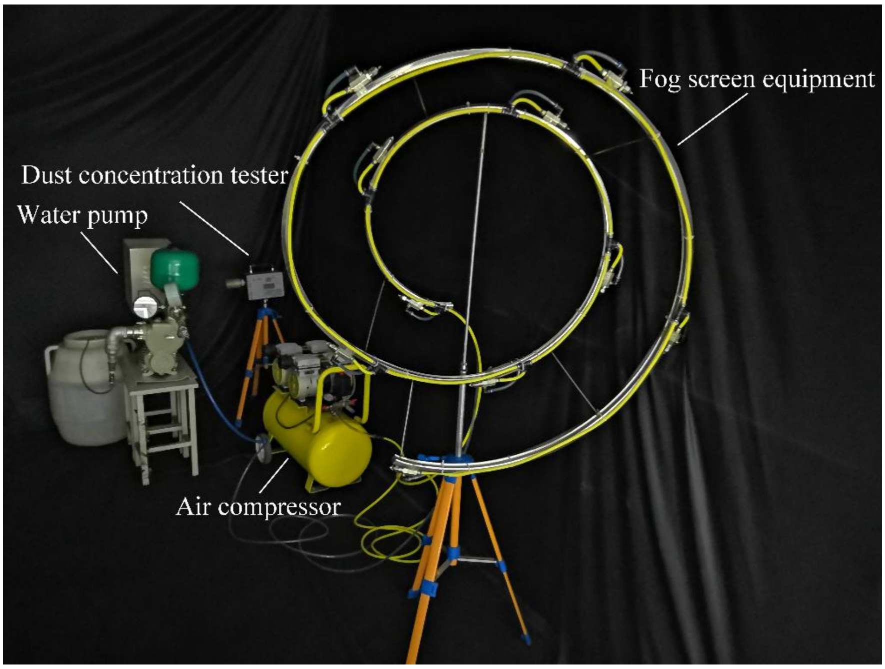

2. The images has been resized appropriately in the manuscript.

3. The size of the formulae in this manuscript has been appropriately resized.

4. The font size of the title of the manuscript has been adjusted to the font size specified in this journal.

5. By simulating the airflow direction from a different number of nozzles in different positions, the simulation results show that when the nozzles are arranged in an arc and the injection direction is tangent to the arc, the jet airflow of each nozzle will interact with each other and make the airflow direction shift to the arc direction.The preliminary demonstration of an important reason for the spiral fog screen produced by the device provides a theoretical basis for the subsequent spray simulation and testing of a multilayer spiral fog screen device.

6. The multilayer spiral fog screen dust removal device uses spiral air flow to break the sprayed

water mist more fully, further improves the atomization effect and the flow speed of the water mist, expands the effective dust isolation range. The spiral air flow forms a negative pressure zone where the dust is drawn into the spiral air flow, increasing the chances of the droplets catching the dust. This device can form a multilayer spiral fog curtain in the roadway to stop dust diffusion and has a more prominent dust removal and dust isolation effect than the traditional spray method. The advantages of these multilayer spiral mist screen devices over conventional spray devices have been added to the section 5.4.2 of the manuscript.

Response to Reviewer#2

1. Page numbers and line numbers have been added to the manuscript.
2. The device has been tested in a real mine environment.
3. Thank you for your valuable suggestions and we will focus on the prediction of dust removal efficiency through numerical simulations in future studies.
4. An explanation of the innovation of the scholars' work has been added to the introduction.
5. A nomenclature table has been added to the section 5.4.1 of the manuscript, with additions and corrections to the explanation of variables in the formulae.
6. Fig 10、5 and 6 has been replaced with a clear picture in this manuscript

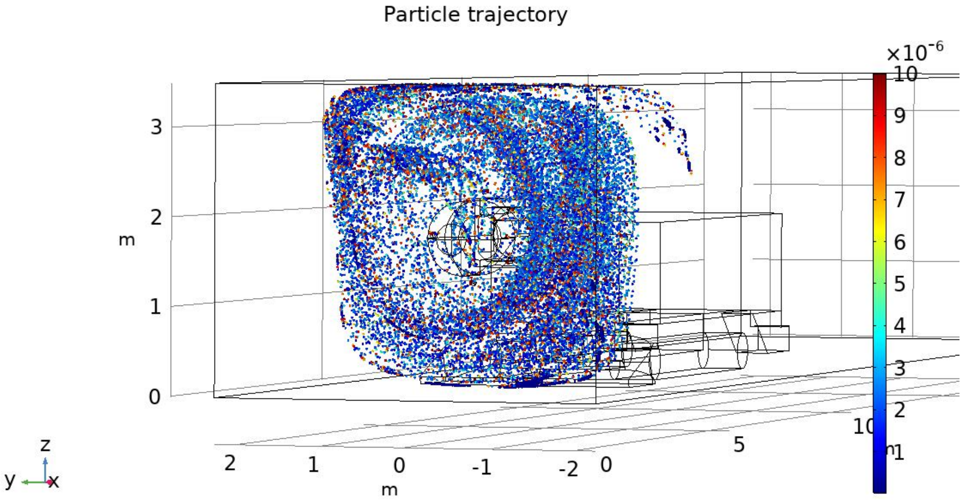

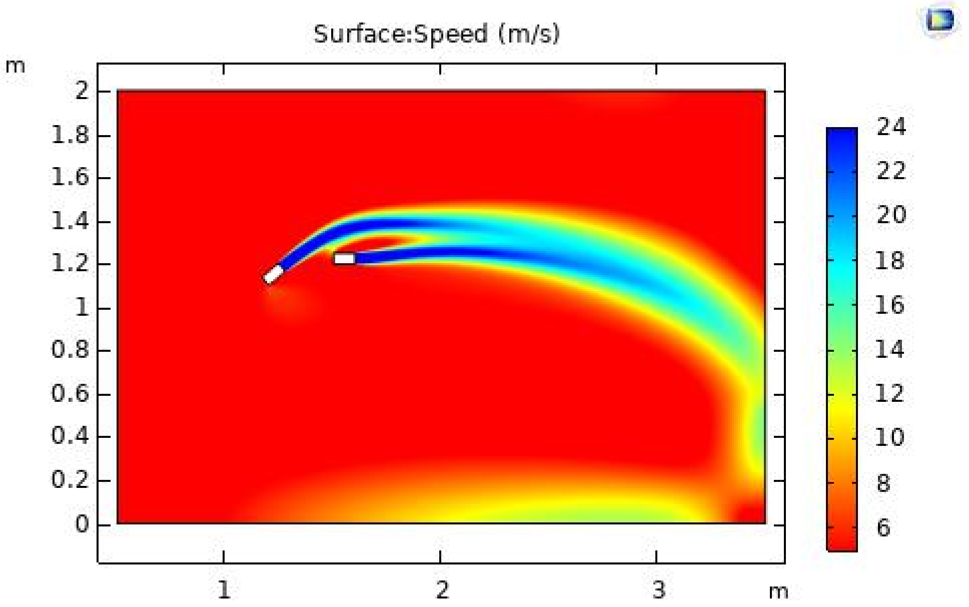

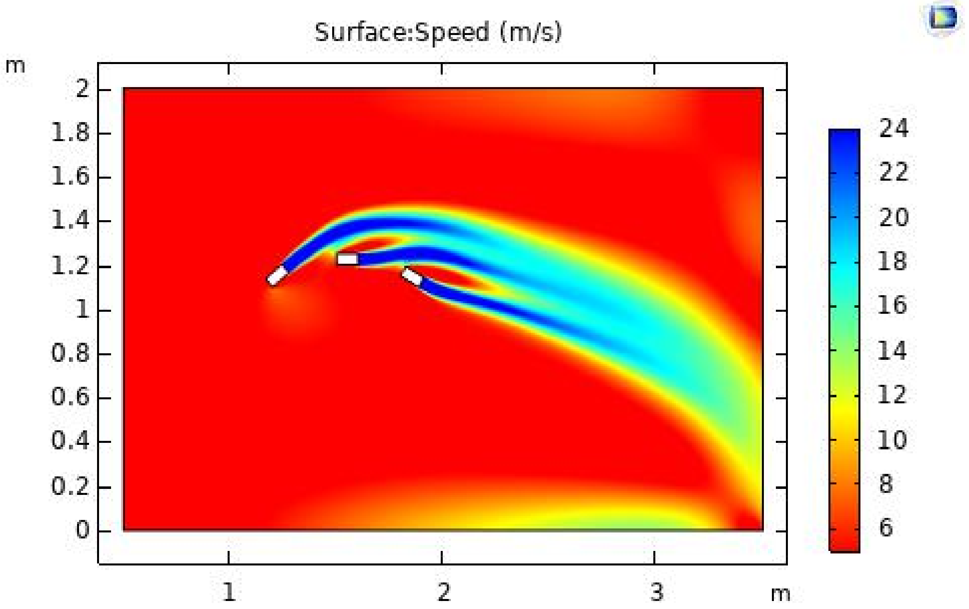

7. Equation numbers have been added to some of the equations in the section 3.2.
8. Reference numbers have been inserted in the text after " author’s name et al.".
9. The same format ("Fig.") has been used in the text to refer to the figures.
10. A space has been used before the first bracket of all in-text citations.
11. The font has been corrected for certain parts of the manuscript where the font differs from the rest of the text (such as references, some tables, the first paragraph of the conclusion, equation numbers and etc.).
12. The rationale of the references cited has been added to the first paragraph of the section 3 of the manuscript, where the references cited are relevant to the content of the study.
13. After all the headline numbers, the punctuation symbol has been added， and some headings beginning with lowercase letters have been corrected.
